# A new species of *Leptobrachella* (Anura, Megophryidae) from Guizhou Province, China

**DOI:** 10.3897/zookeys.923.47172

**Published:** 2020-04-01

**Authors:** Tao Luo, Ning Xiao, Kai Gao, Jiang Zhou

**Affiliations:** 1 State Engineering Tecenology Instiiute For Karst Desertification Control School of Karst Science, Guizhou Normal University, Guiyang 550001, Guizhou, China Guizhou Normal University Guiyang China; 2 Guiyang Nursing Vocational College, Guiyang, Guizhou, 550003, China Guiyang Nursing Vocational College Guiyang China; 3 Ministry of Education Key Laboratory for Biodiversity and Ecological Engineering, College of Life Sciences, Beijing Normal University, Beijing 100875, China Beijing Normal University Beijing China

**Keywords:** *Leptobrachella suiyangensis* sp. nov., mitochondrial DNA, morphology, Southwest China

## Abstract

This study describes a new species of the genus *Leptobrachella*, *Leptobrachella suiyangensis***sp. nov.** from the Huoqiuba Nature Reserve, Suiyang County, Guizhou Province, China, based on morphological data and phylogenetic analyses (16S rRNA mtDNA). The new species can be distinguished from other congeners by the molecular divergence and by a combination of morphological characters, including body size, dorsal and ventral patterns, dorsal skin texture, size of the pectoral and femoral glands, degree of webbing and fringing on the toes and fingers, dorsum coloration, and iris coloration in life. Currently, the genus *Leptobrachella* contains 75 species, 21 of which are found in China, including seven species reported from Guizhou Province. The uncorrected sequence divergence percentage between *Leptobrachella suiyangensis***sp. nov.** and all homologous DNA sequences available for the 16S rRNA gene was found to be >4.7%. The new record of the species and its relationships with others in the same genus imply that species distribution, habitat variation, environmental adaptation, and diversity of the genus *Leptobrachella* in southwest China need to be further investigated.

## Introduction

The genus *Leptolalax* Dubois, 1983 in the family Megophryidae Bonaparte, 1850 is regarded to be closely associated with the genus *Leptobrachella* Smith, 1925 and has been assigned as a synonym of the genus *Leptobrachella* based on a large-scale molecular analysis (Chen et al. 2018). The genus *Leptobrachella* is now considered to contain 74 species. The genus is widely distributed from southwestern China to northeastern India and Myanmar (Fei et al. 2012; Frost 2019), extending to mainland Indochina, peninsular Malaysia, and the islands of Borneo (Rowley et al. 2016, 2017a; Yang et al. 2016; Yuan et al. 2017; Wang et al. 2018; Nguyen et al. 2018). Currently, 20 species of this genus are known from China. They are: *Leptobrachella alpina* (Fei, Ye & Li, 1990) and *L. bourreti* (Dubois, 1983) from Yunnan and Guangxi; *L. eos* (Ohler, Wollenberg, Grosjean, Hendrix, Vences, Ziegler & Dubois, 2011) and *L. nyx* (Ohler, Wollenberg, Grosjean, Hendrix, Vences, Ziegler & Dubois, 2011) from Yunnan; *L. laui* (Sung, Yang & Wang, 2014) and *L. yunkaiensis* Wang, Li, Lyu & Wang, 2018 from southern Guangdong, including Hong Kong; *L. liui* (Fei & Ye, 1990) from Fujian, Jiangxi, Guangdong, Guangxi, Hunan, and Guizhou; *L. oshanensis* (Liu, 1950) from Gansu, Sichuan, Chongqing, Guizhou, and Hubei; *L. purpuraventra* Wang, Li, Li, Chen & Wang, 2019 and *L. bijie* Wang, Li, Li, Chen & Wang, 2019 from Guizhou; *L. purpurus* (Yang, Zeng & Wang, 2018), *L. pelodytoides* (Boulenger, 1893), *L. tengchongensis* (Yang, Wang, Chen & Rao, 2016) and *L. yingjiangensis* (Yang, Zeng & Wang, 2018) from Yunnan; *L. ventripunctata* (Fei, Ye & Li, 1990) from Guizhou and Yunnan; *L. mangshanensis* (Hou, Zhang, Hu, Li, Shi, Chen, Mo & Wang, 2018) from southern Hunan, and *L. sungi* (Lathrop, Murphy, Orlov & Ho, 1998), *L. maoershanensis* (Yuan, Sun, Chen, Rowley & Che, 2017), *L. shangsiensis* Chen, Liao, Zhou & Mo, 2019, and *L. wuhuangmontis* Wang, Yang & Wang, 2018 from Guangxi (Sung et al. 2014; Yang et al. 2016, 2018; Yuan et al. 2017; Wang et al. 2018, 2019; Hou et al. 2018; Chen et al. 2018, 2019; Wang et al. 2019; AmphibiaChina 2019).

**Figure 1.**
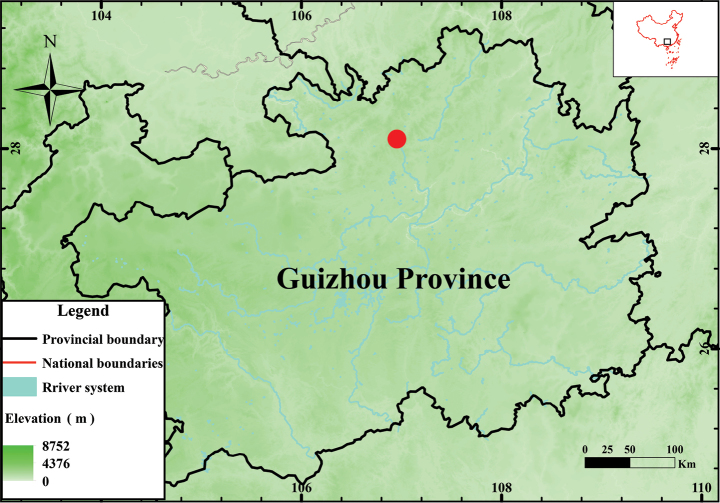
Collection locality (red circle) of *Leptobrachella suiyangensis* sp. nov. from Suiyang County, Guizhou province, China used in this study.

During a field survey in June 2018 in a montane evergreen forest, Suiyang County, Guizhou Province (Fig. 1), we collected three different species of the family Megophryidae co-occurring in this small-fragmented forest. The specimens could be morphologically separated from one another. Subsequent studies based on morphological and molecular data indicated that two of the three could be classified as *Megophrys minor* Stejneger and *M. spinata* Liu and Hu, while the third population, differing significantly from the other two, was further analyzed via morphological characters. Subsequent 16S rRNA sequences from these specimens revealed that the collection represented distinct evolving lineages and belong to the genus *Lepobrachella*. Combining morphological characters, acoustic data, and molecular divergence, we described the specimens as a new species.

## Materials and methods

### Sampling

Eight specimens collected from the aforementioned area (Fig. 1) were euthanized with chlorobutanol solution and fixed in 10% formalin for 24 h, and then stored in 75% ethanol. Liver and muscular tissues were taken before fixing and preserved in 95% alcohol at -20 °C. All of the specimens are kept at the College of Life Sciences, Guizhou Normal University (GZNU), Guiyang City, Guizhou Province, China.

### DNA Extraction, PCR and sequencing

DNA samples were extracted from muscular tissues with a DNA extraction kit (Tiangen Biotech (Beijing) Co. Ltd). The mitochondrial gene and 16S ribosomal RNA gene (16S rRNA) were sequenced (951bp). The fragmented genes were ampliﬁed with primer pairs L3975 (5'-CGCCTGTTTACCAAAAACAT-3') and H4551 (5'-CCGGTCTGAACTCAGATCACGT-3') for 16S rRNA (Simon et al. 1994). PCR ampliﬁcations were performed in a 20 μl reaction volume with the following cycling conditions: an initial denaturing step at 95 °C for five min; 35 cycles of denaturing at 95 °C for 40 s, annealing at 53 °C for 40 s and extending at 72 °C for 1 min, followed by a ﬁnal extending step of 72 °C for 10 min. PCR products were puriﬁed with spin columns. The purified products were sequenced with both forward and reverse primers using a BigDye Terminator Cycle Sequencing Kit according to the guidelines of the manufacturer. The products were sequenced on an ABI Prism 3730 automated DNA sequencer at Shanghai Majorbio Bio-pharm Technology Co. Ltd. All sequences have been deposited in GenBank (Table 1). For molecular analyses, a total of 77 sequences (74 sequences downloaded from GenBank and three our new sequences) from 55 species of the genus *Leptobrachella* were used, including one undescribed species from C hina, that is, the populations from Huoqiuba Nature Reserve, Suiyang County, and Guizhou Province. Three species which sequences downloaded from GenBank are used as outgroups (*Leptobrachium huashen* Fei & Ye, 2005, Leptobrachium cf. chapaense (Bourret, 1937) and *Megophrys major* Boulenger, 1908 (Chen et al. 2018; Wang et al. 2019; Table 1).

**Table 1.** Localities and voucher data for all specimens used in this study.

ID	Species	Locality	Voucher no.	GenBank no.
1	*Leptobrachella suiyangensis* sp.nov.	Suiyang County, Guizhou, China	GZNU20180606002	MK829648
2	*Leptobrachella suiyangensis* sp.nov.	Suiyang County, Guizhou, China	GZNU20180606005	MK829649
3	*Leptobrachella suiyangensis* sp.nov.	Suiyang County, Guizhou, China	GZNU20180606006	MK829650
4	*Leptobrachella aerea*	Vilabuly, Savannakhet, Laos	NCSM 76038	MH055809
5	*Leptobrachella aerea*	Phong Nha-Ke Bang, Quang Binh, Vietnam	RH60165	JN848437
6	*Leptobrachella alpina*	Huangcaoling, Yunnan, China	KIZ046816	MH055866
7	*Leptobrachella applebyi*	Song Thanh Nature Reserve, Quang Nam, Vietnam	AMS R171704	HM133598
8	*Leptobrachella baluensis*	Tambunan, Sabah, Borneo, Malaysia	SP 21604	LC056792
9	*Leptobrachella bidoupensis*	Bidoup, Lam Dong, Vietnam	NCSM 77321	HQ902883
10	*Leptobrachella bijie*	Zhaozishan Nature Reserve, Bijie City, Guizhou, China	SYS a007313/CIB110002	MK414532
11	*Leptobrachella bijie*	Zhaozishan Nature Reserve, Bijie City, Guizhou, China	SYS a007314	MK414533
12	*Leptobrachella botsfordi*	Fansipan, Lao Cai, Vietnam	AMS R 176540	MH055952
13	*Leptobrachella bourreti*	Sapa, Lao Cai, Vietnam	1999.566	KR827860
14	*Leptobrachella brevicrus*	Gunung Mulu National Park, Sarawak, Malaysia	UNIMAS 8957	KJ831303
15	*Leptobrachella dringi*	Gunung Mulu, Malaysia	KUHE:55610	AB847553
16	*Leptobrachella eos*	Boun Tay, Phongsaly, Laos	NCSM 80551	MH055887
17	*Leptobrachella eos*	Zhushihe, Yunnan, China	SYS a003959	MH055888
18	*Leptobrachella firthi*	Ngoc Linh Nature Reserve, Kon Tum, Vietnam	AMS: R 176506	JQ739207
19	*Leptobrachella fritinniens*	Gunung Mulu, Malaysia	KUHE55371	AB847557
20	*Leptobrachella gracilis*	Gunung Mulu, Malaysia	KUHE55624	AB847560
21	*Leptobrachella hamidi*	Bukit Lanjan, Selangor, Malaysia	KUHE17545	AB969286
22	*Leptobrachella heteropus*	Larut, Perak, Malaysia	KUHE15487	AB530453
23	*Leptobrachella isos*	Gia Lai, Vietnam	AMS R 176469	KT824767
24	*Leptobrachella itiokai*	Mulu NP, Sarawak, Borneo, Malaysia	KUHE 55845	LC137802
25	*Leptobrachella juliandringi*	Mulu NP, Sarawak, Borneo, Malaysia	KUHE 55333	LC056780
26	*Leptobrachella kajangensis*	Tioman, Malaysia	LSUHC 4431	LC202001
27	*Leptobrachella kecil*	Cameron, Malaysia	KUHE 52440	LC202004
28	*Leptobrachella khasiorum*	Khasi Hills, Meghalaya, India	SDBDU 2009.329	KY022303
29	*Leptobrachella liui*	Wuyi Shan, Fujian, China	SYS a001597	KM014547
30	*Leptobrachella liui*	Wuyi Shan, Fujian, China	ZYCA907	MH055908
31	*Leptobrachella laui*	Shenzhen, Guangdong, China	SYS a002450	MH055904
32	*Leptobrachella laui*	Shenzhen, Guangdong, China	SYS a001515	KM014545
33	*Leptobrachella macrops*	Phu Yen, Vietnam	ZMMU-A5823	MG787993
34	*Leptobrachella mangshanensis*	Mangshan, Hunan, China	MSZTC201701	MG132196
35	*Leptobrachella mangshanensis*	Mangshan, Hunan, China	MSZTC201702	MG132197
36	*Leptobrachella maoershanensis*	Mao’er Shan, Guangxi, China	KIZ07614	MH055927
37	*Leptobrachella maoershanensis*	Mao’er Shan, Guangxi, China	KIZ027236	MH055928
38	*Leptobrachella marmorata*	Borneo, Malaysia	KUHE53227	AB969289
39	*Leptobrachella maura*	Borneo, Malaysia	SP21450	AB847559
40	*Leptobrachella melanoleucus*	Surat Thani, Thailand	KUHE:23845	LC201999
41	*Leptobrachella melica*	Cambodia, Ratanakiri	MVZ258198	HM133600
42	*Leptobrachella minimus*	Doi Chiang Dao, Chiangmai, Thailand	THNHM07418	JN848402
43	*Leptobrachella minimus*	Doi Suthep, Thailand	KUHE:19201	LC201981
44	*Leptobrachella mjobergi*	Gading NP, Sarawak, Borneo, Malaysia	KUHE:47872	LC056787
45	*Leptobrachella nahangensis*	Na Hang Nature Reserve, Tuyen Quang, Vietnam	ROM 7035	MH055853
46	*Leptobrachella nahangensis*	Na Hang, Tuyen Quang, Vietnam	ZMMU-NAP-02259	MH055854
47	*Leptobrachella nyx*	Ha Giang, Vietnam	ROM 36692	MH055816
48	*Leptobrachella oshanensis*	Emei Shan, Sichuan, China	KIZ025776	MH055895
49	*Leptobrachella oshanensis*	Emei Shan, Sichuan, China	Tissue ID: YPX37492	MH055896
50	*Leptobrachella pallida*	Vietnam: Lam Dong	UNS00511	KU530190
51	*Leptobrachella parva*	Mulu National Park, Sarawak, Malaysia	KUHE:55308	LC056791
52	*Leptobrachella petrops*	Cham Chu Nature Reserve, Tuyen Quang, Vietnam	VNMN:2016 A.06	KY459998
53	*Leptobrachella picta*	Borneo, Malaysia	UNIMAS 8705	KJ831295
54	*Leptobrachella pluvialis*	Fansipan, Lao Cai, Vietnam	ROM 30685	MH055843
55	*Leptobrachella pluvialis*	Sapa, Lao Cai, Vietnam	ZMMU-A-5222-02262	MH055844
56	*Leptobrachella puhoatensis*	Pu Hu, Thanh Hoa, Vietnam	VNMN:2016 A.23	KY849587
57	*Leptobrachella purpura*	Yingjiang, Yunnan Province, China	SYS a006530	MG520354
58	*Leptobrachella purpura*	Yingjiang, Yunnan Province, China	SYS a006531	MG520355
59	*Leptobrachella purpuraventra*	Wujing Nature Reserve, Bijie City, Guizhou, China	SYS a007081	MK414517
60	*Leptobrachella purpuraventra*	Wujing Nature Reserve, Bijie City, Guizhou, China	SYS a007277/CIB110003	MK414518
61	*Leptobrachella pyrrhops*	Lam Dong, Vietnam	ZMMU A-5208	KP017575
62	*Leptobrachella sabahmontana*	Borneo, Malaysia	BORNEENSIS 12632	AB847551
63	*Leptobrachella shangsiensis*	Guangxi, China	NHMG1401032	MK095460
64	*Leptobrachella shangsiensis*	Guangxi, China	NHMG1401033	MK095461
65	*Leptobrachella solus*	Hala-Bala, Thailand	KUHE:23261	LC202007
66	*Leptobrachella solus*	Tam Dao, Vinh Phuc, Vietnam	ROM 20236	MH055858
67	*Leptobrachella tengchongensis*	Gaoligong Shan, Yunnan, China	SYS a004598	KU589209
68	*Leptobrachella tengchongensis*	Gaoligong Shan, Yunnan, China	SYS a003766	MH055897
69	*Leptobrachella ventripunctatus*	Zhushihe, Yunnan, China	SYS a004536	MH055831
70	*Leptobrachella wuhuangmontis*	Mt. Wuhuang, Pubei County, Guangxi, China	SYS a003485	MH605577
71	*Leptobrachella wuhuangmontis*	Mt. Wuhuang, Pubei County, Guangxi, China	SYS a003486	MH605578
72	*Leptobrachella yingjiangensis*	Yingjiang, Yunnan, China	SYS a006533	MG520350
73	*Leptobrachella yingjiangensis*	Yingjiang, Yunnan, China	SYS a006532	MG520351
74	*Leptobrachella yunkaiensis*	Dawuling Forest Station, Maoming City, Guangdong, China	SYS a004663	MH605584
75	*Leptobrachella yunkaiensis*	Dawuling Forest Station, Maoming City, Guangdong, China	SYS a004664 / CIB107272	MH605585
76	*Leptobrachella zhangyapingi*	Chiang Mai, Thailand	KIZ07258	MH055864
77	*Leptobrachella zhangyapingi*	Pang Num Poo, Chiang Mai Province,Thailand	JK-2013	JX069979
78	*Leptobrachium huashen*	Yunnan, China	KIZ049025	KX811931
79	Leptobrachium cf. chapaense	Sapa, Lao Cai, Vietnam	AMS R 171623	KR018126
80	*Megophrys major*	Kon Tum, Vietnam	AMS R 173870	KY476333

### Phylogenetic analyses

All sequences were aligned by MUSCLE v. 3.6 with the default settings (Edgar 2004). Trimming with the gaps partially deleted was performed in MEGA 7.0 (Kumar et al. 2016), while within high variable regions, all gaps were removed.

Phylogenetic trees were constructed with both Maximum Likelihood (ML) and Bayesian Inference (BI). The ML was conducted in IQ-TREE (Nguyen et al. 2015) with 2000 ultrafast bootstrapping (Hoang et al. 2018) and was performed until a correlation coefficient of at least 0.99 was reached. The BI was performed in MrBayes v. 3.2.1 (Ronquist et al. 2012), and the best-fit model was obtained by the Akaike Information Criterion (AIC) computed with PartitionFinder 2 (Lanfear et al. 2016), resulting in the best-fitting nucleotide substitution models of GTR + I + G with for BI and ML analysis. Two independent processes were conducted for 10 million generations, sampling every 1000, with four independent chains and a burn-in of 25%. Convergence was assessed referring to the criteria of all parameters having reached stationarity and having obtained satisfactory effective sample sizes (>200) using Tracer v. 1.6. (Rambaut et al. 2014). Nodes in the trees were considered well supported when Bayesian posterior probabilities (BPP) were ≥0.95 and ML ultrafast bootstrap values (UFB) was ≥95 % (Chen et al. 2018; Hoang et al. 2018). Uncorrected *p*-distances based on 16S rRNA were calculated in MEGA v. 7.0 (Kumar et al. 2016).

### Morphological and morphometric analyses

Morphometric data were taken from eight of most well-preserved adult specimens. Measurements were recorded to the nearest 0.1 mm (Watters et al. 2016) with digital calipers following the methods of Fei et al. (2009) and Rowley et al. (2013). These measurements were as follows:

**SVL** snout-vent length (from tip of snout to vent)

**HDL** head length (from tip of snout to rear of jaws)

**HDW** head width (head width at commissure of jaws)

**SNT** snout length (from tip of snout to the anterior corner of the eye)

**EYE** eye diameter (diameter of the exposed portion of the eyeballs)

**IOD** interorbital distance (minimum distance between upper eyelids)

**IND** internasal distance (distance between nares)

**UEW** upper eyelid width (measured as the greatest width of the upper eyelid)

**NEL** nostril-eyelid length (distance from nostril to eyelid)

**NSL** nostril-snout length (distance from nostril to snout)

**TMP** tympanum diameter (horizontal diameter of tympanum)

**TEY** tympanum-eye distance (distance from anterior edge of tympanum to posterior corner of eye)

**TIB** tibia length (distance from knee to heel)

**ML** manus length (distance from tip of third digit to proximal edge of inner palmar tubercle)

**LAHL** length of the lower arm and hand (distance from tip of the third finger to elbow)

**HLL** hindlimb length (distance from tip of fourth toe to vent)

**FOT** foot length (from proximal edge of the inner metatarsal tubercle to the tip of the fourth toe)

Sex was determined by direct observation of calls in life, the presence of internal vocal sac openings, and the presence of eggs in the abdomen through external inspection. Comparative morphological data of *Leptobrachella* species were obtained from the references listed in Table 2. Due to the high likelihood of undiagnosed diversity within the genus (Rowley et al. 2016; Yang et al. 2016), where available, we relied on examination of topotypic material and/or original species descriptions.

**Table 2.** Obtained references of 74 known congeners of the genus *Leptobrachella*, respectively.

ID	Leptobrachella species	Literature obtained
1	*L. aerea* (Rowley, Stuart, Richards, Phimmachak & Sivongxay, 2010)	Rowley et al. 2010c
2	*L. alpina* (Fei, Ye & Li, 1990)	Fei et al. 2009
3	*L. applebyi* (Rowley & Cao, 2009)	Rowley and Cao 2009
4	*L. arayai* (Matsui, 1997)	Matsui 1997
5	*L. ardens* (Rowley, Tran, Le, Dau, Peloso, Nguyen, Hoang, Nguyen & Ziegler, 2016)	Rowley et al. 2016
6	*L. baluensis* Smith, 1931	Dring 1983; Eto et al. 2016
7	*L. bidoupensis* (Rowley, Le, Tran & Hoang, 2011)	Rowley et al. 2011
8	*L. bijie* Wang, Li, Li, Chen & Wang, 2019	Wang et al. 2019
9	*L. bondangensis* Eto, Matsui, Hamidy, Munir & Iskandar, 2018	Eto et al. 2018
10	*L. botsfordi* (Rowley, Dau & Nguyen, 2013)	Rowley et al. 2013
11	*L. bourreti* (Dubois, 1983)	Ohler et al. 2011
12	*L. brevicrus* Dring, 1983	Dring 1983; Eto et al. 2015
13	*L. crocea* (Rowley, Hoang, Le, Dau & Cao, 2010)	Rowley et al. 2010a
14	*L. dringi* (Dubois, 1987)	Inger et al. 1995; Matsui and Dehling 2012
15	*L. eos* (Ohler, Wollenberg, Grosjean, Hendrix, Vences, Ziegler & Dubois, 2011)	Ohler et al. 2011
16	*L. firthi* (Rowley, Hoang, Dau, Le & Cao, 2012)	Rowley et al. 2012
17	*L. fritinniens* (Dehling & Matsui, 2013)	Dehling and Matsui 2013
18	*L. fuliginosa* (Matsui, 2006)	Matsui 2006
19	*L. fusca* Eto, Matsui, Hamidy, Munir & Iskandar, 2018	Eto et al. 2018
20	*L. gracilis* (Günther, 1872)	Günther 1872; Dehling 2012b
21	*L. hamidi* (Matsui, 1997)	Matsui 1997
22	*L. heteropus* (Boulenger, 1900)	Boulenger 1900
23	*L. isos* (Rowley, Stuart, Neang, Hoang, Dau, Nguyen & Emmett, 2015)	Rowley et al. 2015a
24	*L. itiokai* Eto, Matsui & Nishikawa, 2016	Eto et al. 2016
25	*L. juliandringi* Eto, Matsui & Nishikawa, 2015	Eto et al. 2015
26	*L. kajangensis* (Grismer, Grismer & Youmans, 2004)	Grismer et al. 2004
27	*L. kalonensis* (Rowley, Tran, Le, Dau, Peloso, Nguyen, Hoang, Nguyen & Ziegler, 2016)	Rowley et al. 2016
28	*L. kecil* (Matsui, Belabut, Ahmad & Yong, 2009)	Matsui et al. 2009
29	*L. khasiorum* (Das, Tron, Rangad & Hooroo, 2010)	Das et al. 2010
30	*L. lateralis* (Anderson, 1871)	Anderson 1871; Humtsoe et al. 2008
31	*L. laui* (Sung, Yang & Wang, 2014)	Sung et al. 2014
32	*L. liui* (Fei & Ye, 1990)	Fei et al. 2009; Sung et al. 2014
33	*L. macrops* (Duong, Do, Ngo, Nguyen & Poyarkov, 2018)	Duong et al. 2018
34	*L. maculosa* (Rowley, Tran, Le, Dau, Peloso, Nguyen, Hoang, Nguyen & Ziegler, 2016)	Rowley et al. 2016
35	*L. mangshanensis* (Hou, Zhang, Hu, Li, Shi, Chen, Mo & Wang, 2018)	Hou et al. 2018
36	*L. maoershanensis* (Yuan, Sun, Chen, Rowley & Che, 2017)	Yuan et al. 2017
37	*L. marmorata* (Matsui, Zainudin & Nishikawa, 2014)	Matsui et al. 2014b
38	*L. maura* (Inger, Lakim, Biun & Yambun, 1997)	Inger et al. 1997
39	*L. melanoleuca* (Matsui, 2006)	Matsui 2006
40	*L. melica* (Rowley, Stuart, Neang & Emmett, 2010)	Rowley et al. 2010b
41	*L. minima* (Taylor, 1962)	Taylor 1962; Ohler et al. 2011
42	*L. mjobergi* Smith, 1925	Eto et al. 2015
43	*L. nahangensis* (Lathrop, Murphy, Orlov & Ho, 1998)	Lathrop et al. 1998
44	*L. natunae* (Günther, 1895)	Günther 1895
45	*L. nokrekensis* (Mathew & Sen, 2010)	Mathew and Sen 2010
46	*L. nyx* (Ohler, Wollenberg, Grosjean, Hendrix, Vences, Ziegler & Dubois, 2011)	Ohler et al. 2011
47	*L. oshanensis* (Liu, 1950)	Fei et al. 2009
48	*L. pallida* (Rowley, Tran, Le, Dau, Peloso, Nguyen, Hoang, Nguyen & Ziegler, 2016)	Rowley et al. 2016
49	*L. palmata* Inger & Stuebing, 1992	Inger and Stuebing 1992
50	*L. parva* Dring, 1983	Dring 1983
51	*L. pelodytoides* (Boulenger, 1893)	Boulenger 1893; Ohler et al. 2011
52	*L. petrops* (Rowley, Dau, Hoang, Le, Cutajar & Nguyen, 2017)	Rowley et al. 2017a
53	*L. picta* (Malkmus, 1992)	Malkmus 1992
54	*L. platycephala* (Dehling, 2012)	Dehling 2012a
55	*L. pluvialis* (Ohler, Marquis, Swan & Grosjean, 2000)	Ohler et al. 2000, 2011
56	*L. puhoatensis* (Rowley, Dau & Cao, 2017)	Rowley et al. 2017b
57	*L. purpuraventra* Wang, Li, Li, Chen & Wang, 2019	Wang et al. 2019
58	*L. purpurus* (Yang, Zeng & Wang, 2018)	Yang et al. 2018
59	*L. pyrrhops* (Poyarkov, Rowley, Gogoleva, Vassilieva, Galoyan & Orlov, 2015)	Poyarkov et al. 2015
60	*L. rowleyae* (Nguyen, Poyarkov, Le, Vo, Ninh, Duong, Murphy & Sang, 2018)	Nguyen et al. 2018
61	*L. sabahmontana* (Matsui, Nishikawa & Yambun, 2014)	Matsui et al. 2014a
62	*L. serasanae* Dring, 1983	Dring 1983
63	*L. shangsiensis* Chen, Liao, Zhou & Mo, 2019	Chen et al. 2019
64	*L. sola* (Matsui, 2006)	Matsui 2006
65	*L. sungi* (Lathrop, Murphy, Orlov & Ho, 1998)	Lathrop et al. 1998
66	*L. tadungensis* (Rowley, Tran, Le, Dau, Peloso, Nguyen, Hoang, Nguyen & Ziegler, 2016)	Rowley et al. 2016
67	*L. tamdil* (Sengupta, Sailo, Lalremsanga, Das & Das, 2010)	Sengupta et al. 2010
68	*L. tengchongensis* (Yang, Wang, Chen & Rao, 2016)	Yang et al. 2016
69	*L. tuberosa* (Inger, Orlov & Darevsky, 1999)	Inger et al.1999
70	*L. ventripunctata* (Fei, Ye & Li, 1990)	Fei et al. 2009
71	*L. wuhuangmontis* Wang, Yang & Wang, 2018	Wang et al. 2018
72	*L. yingjiangensis* (Yang, Zeng & Wang, 2018)	Yang et al. 2018
73	*L. yunkaiensis* Wang, Li, Lyu & Wang, 2018	Wang et al. 2018
74	*L. zhangyapingi* (Jiang, Yan, Suwannapoom, Chomdej & Che, 2013)	Jiang et al. 2013

## Results

**Figure 2.**
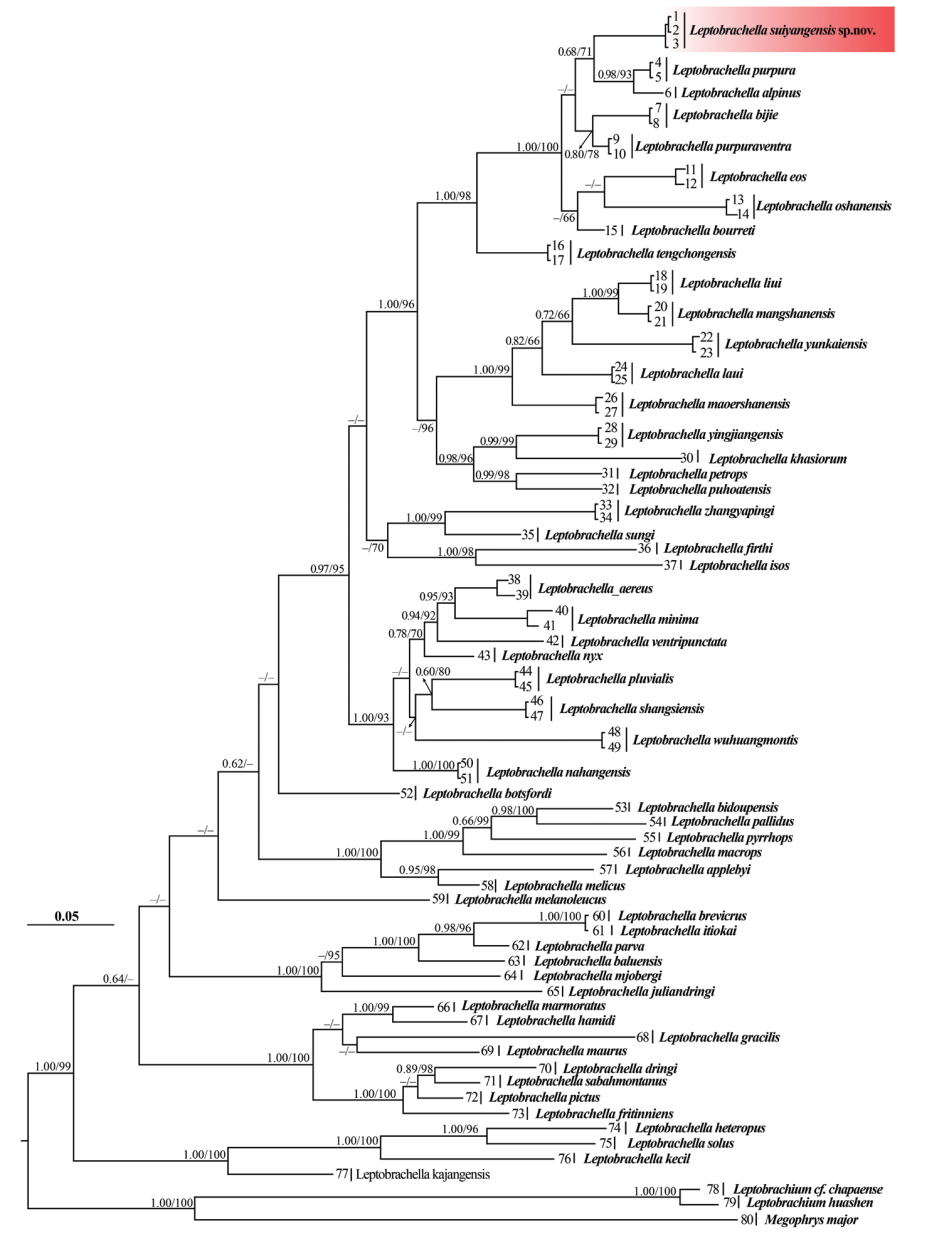
Bayesian inference tree derived from partial DNA sequences of the mitochondrial 16S r RNA gene. Numbers before slashes indicate Bayesian posterior probabilities (displayed >0.60 values), and numbers after slashes are ultrafast bootstrap support for maximum likelihood (2000 replicates) analyses (>60 retained). The symbol “–” represents value below 0.60/60. The scale bar represents 0.05 nucleotide substitutions per site.

Phylogenetic trees from Maximum likelihood (ML) and Bayesian inference (BI) were constructed based on DNA sequences of the mitochondrial 16S rRNA gene with a length of 500 bp. The trees present identical topologies (Fig. 2) with the clustered population of *Leptobrachella* from Huoqiuba Nature Reserve, in which *L. alpina* + *L. purpurus* and the population of *Leptobrachella* from Huoqiuba Nature Reserve show relatively high node supporting values (0.68 in BI and 71% in ML) and exhibit a separate evolving lineage. The smallest pairwise genetic divergence between the population from Suiyang County and all other species of the genus *Leptobrachella* is 4.71%. This indicates that there is substantial genetic divergence between the species in *Leptobrachella* and the specimens from Suiyang County, indicating that this new population can be regarded to be a separate lineage and is valid to be described as a new species as below.

### Taxonomic account

#### Leptobrachella suiyangensis sp. nov.

Taxon classificationAnimaliaAnuraMegophryidae

1238D0CA-8EE7-5DF7-A939-3D20F46EDE06

http://zoobank.org/75EDCF88-0293-40E9-83FE-47785145864C

**Figure 3.**
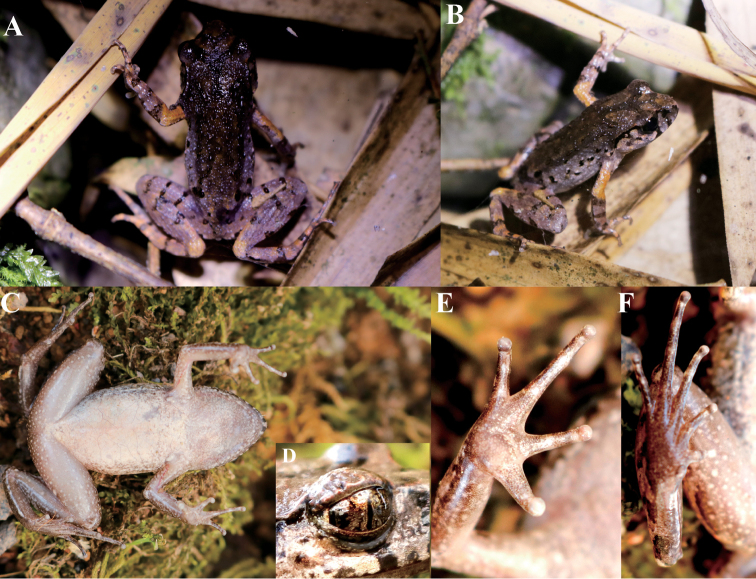
Holotype of *Leptobrachella suiyangensis* sp. nov. (GZNU20180606007) in life. **A** Dorsal view **B** Dorsolateral view **C** Ventral view **D** Right eye shown iris coloration **E** Volar view of the left hand **F** Plantar view of the left foot.

**Figure 4.**
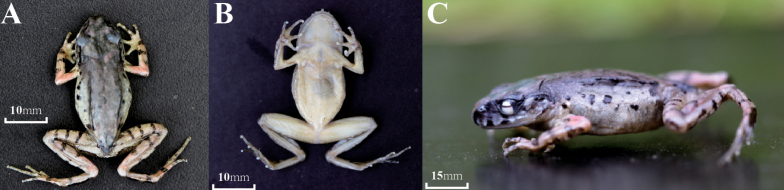
Holotype of *Leptobrachella suiyangensis* sp. nov. (GZNU20180606007) in preservative. **A** Dorsal view **B** Ventral views **C** Lateral views.

Table 3 , Figs 3 , 4

##### Type material.

***Holotype.*** GZNU20180606007, adult male, collected by Tao Luo (TL hereafter) on 7 June 2018 from the Huoqiuba Nature Reserve (28.4805°N, 107.0764°E, 1501 m. a.s.l.; Fig. 1), Suiyang County, Guizhou Province, China.

***Paratypes.*** Five adult males (GZNU20180606002, GZNU20180606005, GZNU20180606006, GZNU20180606008), and three adult females (GZNU20180606001, GZNU20180606003, GZNU20180606004). They were collected from the holotype locality on 6 June 2018.

##### Etymology.

The specific epithet “suiyangensis” refers to the name of the holotype locality, Taibai Town in Suiyang County of Guizohu Province, China. We suggest as its English name “Suiyang Leaf-litter Toad,” and its Chinese name as “Sui Yang Zhang Tu Chan (绥阳掌突蟾)”.

**Table 3.** Measurements (in mm) of the type series of *Leptobrachella suiyangensis***sp.nov.** (H = holotype, P = paratype,M= male, F= female, another abbreviations defined in text).

**Specimen**	**Type status**	**Sex**	**SVL**	**HDL**	**HDW**	**SNT**	**EYE**	**IOD**	**IND**	**UEW**	**NEL**	**NSL**	**TMP**	**TEY**	**TIB**	**HND**	**LAHL**	**HLL**	**FOT**
GZNU20180606007	H	M	28.7	9.9	9.3	4.1	2.4	2.9	2.8	1.6	2.8	1.1	2.1	1.1	13.1	7.2	13.4	43.3	12.5
GZNU20180606008	P	M	29.2	10.5	9.8	4.6	2.8	2.8	2.9	2.1	2.3	1.7	1.2	1.9	13.4	7.0	13.2	43.4	12.9
GZNU20180606002	P	M	29.7	12.1	10.1	5.0	3.9	3.2	3.7	3.1	2.3	1.3	2.3	1.4	13.8	7.1	13.3	44.4	12.3
GZNU20180606005	P	M	29.0	11.8	10.3	4.5	3.3	3.4	3.1	2.0	2.7	1.1	1.9	1.6	13.5	6.5	13.4	41.8	12.9
GZNU20180606006	P	M	29.2	11.4	10.4	4.0	3.8	3.2	3.2	2.6	2.6	2.2	1.8	1.3	13.6	7.4	13.3	42.8	12.6
GZNU20180606001	P	F	32.0	12.6	10.7	4.7	3.7	3.5	3.5	3.0	2.4	1.3	2.6	1.7	15.2	7.1	13.4	44.7	13.9
GZNU20180606003	P	F	30.5	10.3	10.9	4.7	3.7	3.1	3.1	2.2	2.3	1.5	3.5	1.4	15.2	7.4	13.8	45.3	16.6
GZNU20180606004	P	F	33.5	13.1	12.1	4.9	3.6	3.1	3.6	2.8	3.1	1.8	3.8	1.7	17.4	8.1	16.7	53.8	14.4

##### Diagnosis.

The specimens were assigned to the genus *Leptobrachella* on the basis of the following characters: (1) small body size; (2) having an elevated inner metacarpal tubercle; (3) having macro-glands on body (including supra-axillary, femoral and ventrolateral glands); (4) lacking vomerine teeth; (5) having small tubercles on eyelids; (6) anterior tip of snout with whitish vertical bar (Dubois 1983; Matsui 1997, 2006; Lathrop et al. 1998; Delorme et al. 2006; Das et al. 2010). *Leptobrachella suiyangensis* sp. nov. can be distinguished from its congeners by referring to the following characters: (1) small body size (SVL 28.7–29.7 mm in males, 30.5–33.5 mm in females); (2) dorsal skin shagreened, with some of the granules forming longitudinal short skin ridges; (3) tympanum distinctly discernible, slightly concave, with a deep, black, supratympanic line; (4) ventrolateral glands are distinct, forming a dotted line; (5) dorsal surface shagreened and granular, lacking enlarged tubercles or warts, with some of the granules forming short longitudinal folds; (6) flanks with several distinct and large dark blotches; (7) ventral surface of throat grey-white, and surface of chest and belly yellowish creamy-white with marbled texture or with irregular light brown speckling; (8) supra-axillary, femoral, pectoral and ventrolateral glands are distinctly visible; (9) absence of webbing and lateral fringes on fingers, and toes feature rudimentary webbing and a weak lateral fringes; (10) relatively short hindlimbs (TIB/SVL ratio in males 0.46–0.47); (11) longitudinal ridges under the toes are interrupted at the articulations; (12) relative finger lengths I <II < IV < III, relative toe lengths I < II < V < III < IV; (13) dorsum greyish-brown, with small light-orange granules and distinct darker brown markings scattered with irregular light-orange pigmentation, and bicolored iris, coppery orange on the upper half and silver grey on the lower half.

**Description of the holotype.** GZNU20180606007 (adult male), small body size (SVL 28.7 mm); the head length is slightly larger than the head width (HDL/HDW ratio 1.06); the snout is slightly protruding, projecting beyond the margin of the lower jaw; the nostril is between the snout and the eye (NSL/NEL ratio 0.39); the canthus rostralis is gently rounded; the loreal region is slightly concave; the interorbital space is flat; larger (IOD 2.9 mm) than the upper eyelid (1.6 mm in width), and the internarial distance is 2.8 mm; with vertical pupil; snout length is slightly larger than eye diameter (SNT/EYE ratio 1.71); tympanum is distinct and rounded, its diameter (TMP 2.1 mm) is smaller than that of the eye diameter (EYE 2.4 mm) and longer than the tympanum-eye distance (TMP/TEY ratio 1.91); deep black supratympanic line is present; weakly black supratympanic line exists (Fig. 3C); tympanic rim is distinctly elevated relative to the skin of the temporal region; supratympanic ridge is distinct, extending from the eye to the supra-axillary gland; a few indistinct tubercles present on supratympanic ridge; absent vomerine teeth; vocal sac openings is slit-like, located posterior-laterally on the floor of the mouth close to the margins of the mandible; long and wide tongue, with a small shallow notch at the posterior tip.

The tips of the fingers are rounded, slightly swollen; relative finger lengths are presented as: I <II < IV < III; nuptial pad is absent; absent subarticular tubercles (Fig. 3F); a large, round inner palmar tubercle is distinctly separated from a small, round outer palmar tubercle; finger webbing and dermal fringes absent. Toe tips are similar to those of the fingers; the relative toe length is presented as: I <II <V<III <IV; absent subarticular tubercles; distinct dermal ridges present under the 3^rd^ to 5^th^ toes; pronounced larger, oval inner metatarsal tubercle, outer metatarsal tubercle is absent; rudimentary toe webbing; weak lateral fringes present on all toes. Tibia is slightly shorter than half of the snout-vent length (TIB/SVL ratio 0.46); tibiotarsal articulation reaches to the anterior eye; heels meet each other when thighs are appressed at right angles referring to the body.

Dorsal skin is shagreened and scattered with fine and rounded granules, some of the granules forming short longitudinal folds; ventral skin smooth; large pectoral gland, elongated oval, 1.5 mm in length; small femoral gland, rounded, 0.7 mm in diameter, situated on the posteroventral surface of the thigh, closer to tibiotarsal articulation than to the vent; risen supra-axillary gland, 1.3 mm in diameter; ventrolateral gland is distinct as small white dots forming an incomplete line (Fig. 3D).

**Measurements of holotype (in mm).** Holotype: SVL 28.7, HDL 9.9, HDW 9.3, SNT 4.1, EYE 2.4, IOD 2.9, INT 2.8, UEW 1.6, NEL 2.8, NSL 1.1, TMP 2.1, TEY 1.1, TIB 13.1, HND 7.2, LAHL 13.4, HLL 43.3, FOT 12.5.

**Coloration of holotype in life.** Dorsal skin purple-brown; brown-purplish with dark-brown marks between the eyes and the scapular region, which are scattered with some deep yellow-orange granules more concentrated on the upper eyelid (Fig. 3C). A dark brown ϒ-pattern exists between eyes, linked with dark brown W-shaped marks between axillae. Tympanum is light brown-grey; black-brown tubercles present on dorsum of the body and the limb; those on dorsal side are much more distinct and dense; anterior upper lip features distinct blackish-brown patches; transverse dark-brown bars exist on dorsal surface of the limbs two or three (elbow and upper arms are an exception); indistinct black or brown blotches present on the flanks from groin to axilla; elbow and upper arms have no dark bars but with distinct dark-orange coloration; fingers and toes show indistinct brown blotches; a black spot is present on the loreal region; lower edge of the upper drum ridge is prominently black; ventral surface of the throat is grey-white, and surface of chest and belly is yellowish creamy-white, ventral part with distinct or indistinct light brown speckling mixed with marble texture; ventral surface of the thighs is dark grey and scattered with small light white spots. Supra-axillary gland milky yellow; iris is bicolored, coppery orange on the upper half and silver grey on the lower half.

##### Coloration of holotype in preservative.

In preservation, there are dark brown marks on the dorsum and flanks; dorsum of the body and hindlimbs are dark brown, while dorsum of the forelimbs is yellowish brown; transverse bars on the limbs become more distinct, and dark-brown patterns, marks and spots on the back are indistinct; ventral surface of the body is yellowish brown with brown marbling on the sides and chest; orange supra-axillary, femoral, pectoral and ventrolateral glands fade to greyish white.

##### Variations.

**Figure 5.**
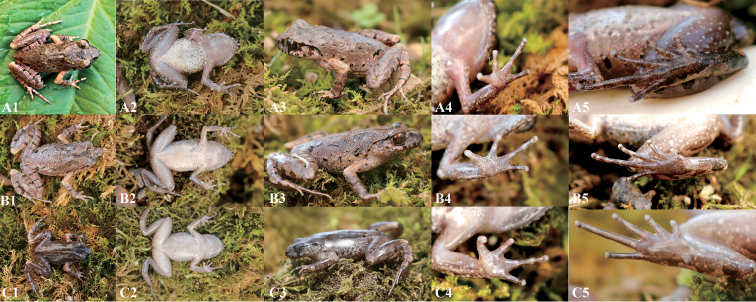
Paratypes of *Leptobrachella suiyangensis* sp. nov. in life. **A** GZNU20180606005, adult male (**A**), (**B**) GZNU20180606002, adult male **C** GZNU20180606003, adult female.

Measurements of the type series are shown in Table 4. Females (mean of SVL (32.0 ± 1.5 mm, *n* = 3) have larger body size than males (mean of SVL 29.2 ± 0.4 mm, *n* = 5) (Table 4). In life (Fig. 5), all paratypes match overall characters of the holotype, except the surface of the belly that is scattered with brown speckling in the holotype (that for females is more distinct; GZNU20180606001). Under the condition of preservation, however, some specimens become slightly darker brown compared to the holotype.

##### Distribution and habitats.

Currently, *Leptobrachella suiyangensis* sp. nov. is known only from its holotype locality, Huoqiuba Nature Reserve, Suiyang County, Guizhou Province, China (Fig. 1). The specimens were collected in a stream (ca 1.5 m in width and ca 10 cm in depth) and from nearby well-preserved bamboo forests (1501 m a.s.l.). During June, males were calling from under bamboo leaves; others perch on or under rocks by the side of the stream.

**Table 4.** Measurements (in mm), and body proportions of *Leptobrachella suiyangensis***sp.nov.** from Suiyang County, Guizhou Province, China.

**Measurements**	**Males Range (mean ± SD), n = 5**	**Females Range (mean ± SD), n = 3**
SVL	28.7–29.7 (29.2 ± 0.4)	30.5–33.5 (32.0 ± 1.5)
HDL	9.9–12.1 (11.1 ± 0.9)	10.3–13.1 (12.0 ± 1.5)
HDW	9.3–10.4 (10.0 ± 0.4)	10.7–12.1 (11.2 ± 0.8)
SNT	4.0–5.0 (4.4 ± 0.4)	4.7–4.9 (4.8 ± 0.1)
EYE	2.4–3.9 (3.2 ± 0.6)	3.6–3.7 (3.7 ± 0.1)
IOD	2.8–3.4 (3.1 ± 0.2)	3.1–3.5 (3.2 ± 0.2)
INT	2.8–3.7 (3.1 ± 0.4)	3.1–3.6 (3.4 ± 0.3)
UEW	1.6–3.1 (2.3 ± 0.6)	2.2–3.0 (2.7 ± 0.4)
NEL	2.3–2.8 (2.5 ± 0.2)	2.3–3.1 (2.6 ± 0.4)
NSL	1.1–2.2 (1.5 ± 0.5)	1.3–1.8(1.5 ± 0.3)
TMP	1.2–2.3 (1.9 ± 0.4)	2.6–3.8 (3.3 ± 0.6)
TEY	1.1–1.9 (1.5 ± 0.3)	1.4–1.7 (1.6 ± 0.2)
TIB	13.1–13.8 (13.5 ± 0.3)	15.2–17.4 (15.9 ± 1.3)
HND	6.5–7.4 (7.0 ± 0.3)	7.1–8.1 (7.5 ± 0.5)
LAHL	13.2–13.4 (13.3 ± 0.1)	13.4–16.7 (14.6 ± 1.8)
HLL	41.8–44.4 (43.1 ± 0.9)	44.7–53.8 (47.9 ± 5.1)
FOT	12.3–12.9 (12.6 ± 0.3)	13.9–16.6 (15.0 ± 1.4)
HDL/HDW	1.06–1.20 (1.12 ± 0.06)	0.90–1.20 (1.07 ± 0.15)
HDL/SVL	0.34–0.41 (0.38 ± 0.03)	0.30–0.40 (0.37 ± 0.06)
SNT/HDL	0.35–0.44 (0.40 ± 0.03)	0.40–0.50 (0.43 ± 0.06)
SNT/EYE	1.05–1.71 (1.41 ± 0.27)	1.30–1.40 (1.33 ± 0.06)
EYE/TMP	1.14–2.33 (1.80 ± 0.45)	1.00–1.40 (1.17 ± 0.21)
EYE/SNT	0.59–0.95 (0.73 ± 0.15)	0.70–0.80 (0.77 ± 0.06)
TMP/EYE	0.43–0.88 (0.59 ± 0.18)	0.70–1.10 (0.93 ± 0.21)
TIB/SVL	0.46–0.47 (0.46 ± 0.01)	0.50
LAHL/SVL	0.45–0.47 (0.46 ± 0.01)	0.40–0.50 (0.47 ± 0.06)
HLL/SVL	1.44–1.51 (1.48 ± 0.03)	1.40–1.60 (1.50 ± 0.10)
TIB/HLL	0.30–0.32 (0.31 ± 0.01)	0.30

### Comparisons

*Leptobrachella suiyangensis* sp. nov. differs from all other species of *Leptobrachella* based on morphological and molecular evidence. Phylogenetically, *L. suiyangensis* sp. nov., *L. alpina* and *L. purpurus* form a clade. Genetically, among this clade, the smallest genetic distance, at 5.49%, is between *L. suiyangensis* sp. nov. and *L. alpina*, and the largest genetic distance is 6.27% (*L. suiyangensis* sp. nov. and *L. purpurus*). Morphologically, the new species can be distinguished from *L. alpina* by having a larger body size of males (28.7–29.7 mm vs 24.0–26.4 mm); having narrower lateral fringes on the toes of the male (vs wide in males); dorsum purple-brown to dark purple-brown or grey-purple ground colour; ventral yellowish creamy-white with marbled texture on the chest and belly or with irregular light-brown speckling (vs almost uniformly gray-brown on dorsal part, ventral nearly immaculately creamy white, brown specking on margins); ventrolateral glands are characterized by small white dots forming an incomplete line (vs small white dots forming a complete line longitudinally); shoulder-gland is orange-yellow (vs white, around gland); head length greater than head width, HDL/HDW ratio 1.12 (vs head length equal to head width, HDL/HDW ratio 1.00). The new species can be distinguished from *L. purpurus* by body size of males (28.7–29.7 mm vs 25.0–27.5 mm); having narrow lateral fringes on the toes of males (vs wide in males); dorsum purple-brown to dark purple-brown or grey-purple ground color, ventral yellowish creamy-white with marbled texture on the chest and belly or with irregular light-brown speckling (vs dorsum coloration purplish brown, ventral side dull white with an indistinct grey dusting); throat immaculate gray (vs throat immaculate pinkish; almost dark orange-yellow on the upper arm (vs upper arms with distinct coppery orange coloration); dark bars on dorsal surface of tibia and tarsus very narrow, especially those on dorsal skin of tarsus (vs relatively broader dark bars on dorsal surface of tibia and tarsus); tibiotarsal articulation reaches to the anterior eye (vs tibiotarsal articulation reaches to posterior corner of the eye); relative length of fingers I <II < IV < III (vs I = II = IV < III).

Compared with the 26 known congeners in the genus *Leptobrachella* found south of the Isthmus of Kra, referring to the presence or absence of supra-axillary and ventrolateral glands, *L. suiyangensis* sp. nov. can be easily distinguished from *L. arayai*, *L. dringi*, *L. fritinniens*, *L. gracilis*, *L. hamidi*, *L. heteropus*, *L. kajangensis*, *L. kecil*, *L. marmorata*, *L. maura*, *L. melanoleuca*, *L. picta*, *L. platycephala*, *L. sabahmontana* and *L. sola*, all of which are lack of supra-axillary and ventrolateral glands (Dubois et al. 2010; Dehling and Matsui 2013; Matsui et al. 2014). As for the comparison referring to the body size, the new species shows a significantly larger body size (SVL, 28.7–29.7 mm in males) than *L. baluensis* (14.9–15.9 mm in males), *L. brevicrus* (17.1–17.8 mm in males), *L. itiokai* (15.2–16.7 mm In males), *L. juliandringi* (17.0–17.2 mm in males), *L. mjobergi* (15.7–19.0 mm in males), *L. natunae* (17.6 mm in one adult male), *L. parva* (15.0–16.9 mm in males), *L. palmata* (14.4–16.8 mm in males), *L. serasanae* (16.9 mm in one adult male), and *Leptobrachella* sp. 3 “baluensis” (15.0–16.0 mm in males).

From the remaining 48 known congeners in the genus *Leptobrachella* found north of the Isthmus of Kra (Table 5) with SVL 28.7–29.7 mm in males and SVL 30.5–33.5 mm in females, *L. suiyangensis* sp. nov. can be distinguished from the larger *L. bourreti* (42.0–45.0 mm in females), *L. eos* (33.1–34.7 mm in males and 40.7 in one female), *L. lateralis* (36.6 mm in females), *L. nahangensis* (40.8 mm in one male), *L. nyx* (37.0–41.0 mm in females), *L. pyrrhops* (30.8–34.3 mm in males), *L. sungi* (48.3–52.7 mm in males and 56.7–58.9 mm in females), *L. tamdil* (32.3 mm in males) and *L. zhangyapingi* (45.8–52.5 mm in males), and from the smaller *L. alpina* (24.0–26.4 mm in males), *L. applebyi* (19.6–22.3 mm in males and 21.7–26.4 mm in females), *L. ardens* (21.3–24.7 mm in males and 24.5 mm in female), *L. bidoupensis* (18.5–25.4 mm in males and 29.2–29.4 mm in females), *L. khasiorum* (24.5–27.3 mm in males), *L. laui* (24.8–26.7 mm in males), *L. maculosa* (24.2–26.6 in males and 27.0 mm in one female), *L. melica* (19.5–22.7 mm in males), *L. maoershanensis* (29.1 mm in one female), *L. petrops* (23.6–27.6 mm in males), *L. pluvialis* (21.3–22.3 mm in males), *L. purpurus* (25.0–27.5 mm in males), *L. rowleyae* (23.1–28.1 mm in males and 27.0–27.8 mm in females), *L. ventripunctata* (25.5–28.0 mm in males), *L. tengchongensis* (23.9–26.0 mm in males and 28.8–28.9 mm in females) and *L. yingjiangensis* (25.7–27.6 mm in males).

**Table 5.** Selected diagnostic characters for species described herein and species in the genus *Leptobrachella* occurring north of the Isthmus of Kra (modified from Rowley et al. 2017; Yuan et al. 2017; Hou et al. 2018; Wang et al. 2018).

**ID**	**Species**	**Males SVL (mm)**	**Black spots on flanks**	**Toes webbing**	**Fringes on toes**	**Ventral coloration**	**Dorsal skin texture**
1	*L. suiyangensis* sp. nov.	28.7–29.7	Yes	Rudimentary	Narrow	Yellowish creamy-white with marble texture chest and belly or with irregular light brown speckling	Shagreen with small granules
2	*L. aerea*	25.1–28.9	No	Rudimentary	Wide	Near immaculate creamy white, brown specking on margins	Finely tuberculate
3	*L. alpinus*	24.0–26.4	Yes	Rudimentary	Wide in males	Creamy-white with dark spots	Relatively smooth, some with small warts
4	*L. applebyi*	19.6–22.3	Yes	Rudimentary	No	Reddish brown with white speckling	Smooth
5	*L. ardens*	21.3–24.7	Yes	No	No	Reddish brown with white speckling	Smooth- finely shagreened
6	*L. bidoupensis*	18.5–25.4	Yes	Rudimentary	Weak	Reddish brown with white speckling	Smooth
7	*L. botsfordi*	29.1–32.6	No	Rudimentary	Narrow	Reddish brown with white speckling	Shagreened
8	*L. bourreti*	28.0–36.2	Yes	Rudimentary	Weak	Creamy white	Relatively smooth, some with small warts
9	*L. crocea*	22.2–27.3	No	Rudimentary	No	Bright orange	Highly tuberculate
10	*L. eos*	33.1–34.7	No	Rudimentary	Wide	Creamy white	Shagreened
11	*L. firthi*	26.4–29.2	No	Rudimentary	Wide in males	Creamy white	Shagreened with fine tubercles
12	*L. fuliginosa*	28.2–30.0	Yes	Rudimentary	Weak	White with brown dusting	Nearly smooth, few tubercles
13	*L. isos*	23.7–27.9	No	Rudimentary	Wide in males	Creamy white with white dusting on margins	Mostly smooth, females more tuberculate
14	*L. kalonensis*	25.8–30.6	Yes	No	No	Pale, speckled brown	Smooth
15	*L. khasiorum*	24.5–27.3	Yes	Rudimentary	Wide	Creamy white	Isolated, scattered tubercles
16	*L. lateralis*	26.9–28.3	Yes	Rudimentary	No	Creamy white	Roughly granular
17	*L. laui*	24.8–26.7	Yes	Rudimentary	Wide	Creamy white with dark brown dusting on margins	Round granular tubercles
18	*L. liui*	23.0–28.7	Yes	Rudimentary	Wide	Creamy white with dark brown spots on chest and margins	Round granular tubercles with glandular folds
19	*L. macrops*	28.0–29.3	Yes	Rudimentary	No	Greyish-violet with white speckling	Roughly granular with larger tubercles
20	*L. maculosa*	24.2–26.6	Yes	No	No	Brown, less white speckling	Dorsum mostly smooth with numerous tiny tubercles
21	*L. mangshanensis*	22.2–27.8	Yes	Rudimentary	Weak	White speckles on throat and belly	Nearly smooth
22	*L. maoershanensis*	25.2–30.4	Yes	Rudimentary	Narrow	Creamy white chest and belly with irregular black spots	Longitudinal folds
23	*L. melica*	19.5–22.7	Yes	Rudimentary	No	Reddish brown with white speckling	Smooth
24	*L. minima*	25.7–31.4	Yes	Rudimentary	No	Creamy white	Smooth
25	*L. nahangensis*	40.8	Yes	Rudimentary	No	Creamy white with light specking on throat and chest	Smooth
26	*L. nokrekensis*	26.0–33.0	Yes	Rudimentary	unknown	Creamy white	Tubercles and longitudinal folds
27	*L. nyx*	26.7–32.6	Yes	Rudimentary	No	Creamy white with white with brown margins	Rounded tubercles
28	*L. oshanensis*	26.6–30.7	Yes	No	No	Whitish with no markings or only small, light grey spots	Smooth with few glandular ridges
29	*L. pallida*	24.5–27.7	No	No	No	Reddish brown with white speckling	Tuberculate
30	*L. pelodytoides*	27.5–32.3	Yes	Wide	Narrow	Whitish	Small, smooth warts
31	*L. petrops*	23.6–27.6	No	No	Narrow	Immaculate creamy white	Highly tuberculate
32	*L. pluvialis*	21.3–22.3	Yes	Rudimentary	No	Dirty white with dark brown marbling	Smooth, flattened tubercles on flanks
33	*L. puhoatensis*	24.2–28.1	Yes	Rudimentary	Narrow	Reddish brown with white dusting	Longitudinal skin ridges
34	*L. purpurus*	25.0–27.5	Yes	Rudimentary	Wide	Dull white with indistinct grey dusting	Shagreen with small tubercles
35	*L. pyrrhops*	30.8–34.3	Yes	Rudimentary	No	Reddish brown with white speckling	Slightly shagreened
36	*L. rowleyae*	23.4–25.4	Yes	No	No	Pinkish milk-white to light brown chest and belly with numerous white speckles	Smooth with numerous tiny tubercles
37	*L. sungi*	48.3–52.7	No or small	Wide	Weak	White	Granular
38	*L. shangsiensis*	24.9–29.4	Yes	Narrow	Narrow	Yellowish creamy-white with marble texture	Smooth
39	*L. tadungensis*	23.3–28.2	Yes	No	No	Reddish brown with white speckling	Smooth
40	*L. tamdil*	32.3	Yes	Wide	Wide	White	Weakly tuberculate
41	*L. tengchongensis*	23.9–26.0	Yes	Rudimentary	Narrow	White with dark brown blotches	Shagreened with small tubercles
42	*L. tuberosa*	24.4–29.5	No	Rudimentary	No	White with small grey spots/streaks	Highly tuberculate
43	*L. ventripunctata*	25.5–28.0	Yes	Rudimentary	No	Chest and belly with dark brown spots	Longitudinal skin ridges
44	*L. wuhuangmontis*	25.6–30.0	Yes	Rudimentary	Narrow	Greyish white mixed by tiny white and black dots	Rough, scattered with dense conical tubercles
45	*L. yingjiangensis*	25.7–27.6	Yes	Rudimentary	Wide	Creamy white with dark brown flecks on chest and margins	Shagreened with small tubercles
46	*L. yunkaiensis*	25.9–29.3	Yes	Rudimentary	Wide	Belly pink with distinct or indistinct speckling	Shagreened with short skin ridges and raised warts
47	*L. zhangyapingi*	45.8–52.5	No	Rudimentary	Wide	Creamy-white with white with brown margins	Mostly smooth with distinct tubercles
48	*L. bijie*	29.0–30.4	Yes	Rudimentary	Narrow	White with distinct nebulous greyish speckling on chest and ventrolateral flanks	Shagreened and granular
49	*L. purpuraventra*	27.3–29.8	Yes	Rudimentary	Narrow	Grey purple with distinct nebulous greyish speckling on chest and ventrolateral flanks	Shagreened and granular

In having irregular, light-brown speckling on the flanks, the new species differs from *L. aerea*, *L. botsfordi*, *L. crocea*, *L. firthi*, *L. isos*, *L. pallida*, *L. petrops* and *L. tuberosa*, all of which lack distinct irregular, light-brown speckling on the flanks. By having rudimentary webbing on the toes, the new species differs from *L. kalonensis*, *L. oshanensis*, *L. pallida*, *L. petrops*, and *L. tadungensis*, all of which lack webbing on the toes; and from *L. pelodytoides*, which has wide webbing on the toes. By having narrow lateral fringes on toes, the new species differs from *L. ardens*, *L. eos*, *L. firthi*, *L. isos*, *L. khasiorum*, *L. laui*, *L. liui*, *L. purpurus*, *L. tamdil*, *L. yingjiangensi*s and *L. yunkaiensis*, all of which have wide lateral fringes on the toes; from *L. bidoupensis*, *L. bourreti*, *L. fuliginosa* and *L. mangshanensis*, all of which have weak lateral fringes on the toes; and from *L. crocea*, *L. kalonensis*, *L. lateralis*, *L. macrops*, *L. minima*, *L. nyx*, *L. oshanensis*, *L. pallida*, *L. pyrrhops*, *L. tadungensis*, *L. tuberosa*, and *L. ventripunctata*, all of which lack lateral fringes on the toes. By having dorsal surface shagreened with small granules, and in lacking enlarge tubercles or warts, the new species differs from *L. applebyi*, *L. bidoupensis, L. kalonensis*, *L. melica, L. minima*, *L. nahangensis*, *L. shangsiensis* and *L. tadungensis*, all of which have the dorsum smooth, and *L. alpina* (dorsum smooth, some with small warts), *L. fuliginosa* (dorsum smooth with fine tubercles), *L. laui* (dorsum with round granular tubercle, lacking skin ridges), *L. liui* (dorsum with round tubercles), *L. macrops* (dorsum roughly granular with large tubercles), *L. maoershanensis* (dorsum smooth with small warts), *L. nokrekensis* (dorsum tubercles and longitudinal folds), *L. pelodytoides* (dorsum with small, smooth warts), *L. puhoatensis* (dorsum longitudinal skin ridges), *L. tuberosa* (dorsum highly tuberculate), *L. yunkaiensis* (dorsum with raised warts), *L. wuhuangmontis* (dorsum rough with conical tubercles), and *L. bijie* and *L. purpuraventra* (dorsum shagreened and granular). By the yellowish creamy-white with marbled chest and belly or with irregular light-brown speckling, the new species differs from *L. alpinus*, *L. applebyi*, *L. ardens*, *L. bidoupensis*, *L. botsfordi* and *L. pyrrhops* (ventral reddish brown with white speckling), *L. aerea* (ventral nearly immaculate creamy-white with brown specking on margins), *L. bijie* (ventral white with distinct nebulous greyish speckling on chest and ventrolateral flanks), *L. crocea* (ventral bright orange), *L. khasiorum, L. nokrekensis* and *L. yingjiangensis* (ventral creamy white), *L. macrops* (ventral greyish-violet with white speckling), *L. puhoatensis* (ventral reddish-brown with white dusting), *L. purpurus* (ventral dull white with indistinct grey dusting), *L. purpuraventra* (ventral grey-purple with distinct nebulous greyish speckling on the chest and ventrolateral flanks), *L. tuberosa* (ventral white with small grey spots and streaks), *L. ventripunctata* (chest and belly with large dark brown spots), *L. wuhuangmontis* (ventral greyish white), and *L. yunkaiensis* (belly pink with speckling). A comparative morphological data (selection) of *Leptobrachella suiyangensis* sp. nov. and 48 recognized *Leptobrachella* species occurring north of the Isthmus of Kra are listed in Table 5.

## Discussion

Phylogenetic analyses based on mitochondrial DNA and nuclear DNA all suggested that the new species belongs to *Leptobrachella* but is separate from its congeners. Genetic distance of the 16S rRNA gene between the new species and its closely related species (*L. bijie*, *L. purpuraventra*, *L. alpina* and *L. purpurus*) was 4.71–6.27%, within the expected range of interspecific divergences in amphibians (Fouquet et al. 2007), and this genetic distance is much higher than between many sister species, of which, most species have been completely recognized as valid species. For example, in *Leptobrachella*, the *p*-distance = 2.35% between *L. purpurus* and *L. alpina*. Finally, a series of morphological characters were found to be different between the new species and its congenerson. All in all, multiple pieces of evidence support the validity of the new species.

The new species described in this study increases the number of species of *Leptobrachella* to 75, with 21 recorded from China (Fei et al. 2012; Sung et al. 2014; Yang et al. 2016, 2018; Yuan et al. 2017; Hou et al. 2018; Wang et al. 2018, 2019; Chen et al. 2018, 2019; Frost 2019). Before the description of the new species herein, only 12 species were recorded from southwest China. This highlights the underestimation of the species diversity of the genus *Leptobrachella*. Additional field surveys are required to understand the true diversity of amphibians in this genus, which will be useful for conservation strategies.

Studies on the taxonomy and phylogeny of the genus *Leptobrachella* were difficult to perform because of the morphological conservativeness of the species; in the field, many species appear to be very similar morphologically, and there exist sympatric species. This likely hinders our understanding of these cryptic species (Ohler et al. 2010; Sung et al. 2014; Yang et al. 2016, 2018; Yuan et al. 2017; Hou et al. 2018; Wang et al. 2018, 2019; Chen et al. 2019). The high species diversity and the degree of endemism indicated that the speciation pattern and sympatry mechanism of species in the genus *Leptobrachella* also need additional investigation.

Currently, to our knowledge, *L. suiyangensis* sp. nov. is restricted to rocky streams in bamboo forests. However, the type locality of *L. suiyangensis* sp. nov. has faced habitat loss and human disturbance, such as artificial grazing and herb collection, which could possibly threaten this species. *Leptobrachella suiyangensis* sp. nov. is range-restricted to Kuankuoshui National Nature Reserve, which borders the nearby Huoqiuba Nature Reserve and is in the eastern Ta-lou Mountains. These areas feature subtropical evergreen broad-leaved forest and evergreen deciduous broad-leaved mixed forest. Thus, it is likely that other populations of *L. suiyangensis* sp. nov. may be discovered in the Kuankuoshui Nature Reserve in the near future.

## Supplementary Material

XML Treatment for Leptobrachella suiyangensis

## References

[B1] Anderson J (1871) A list of the reptilian accession to the Indian Museum, Calcutta from 1865 to 1870, with a description of some new species. Journal of the Asiatic Society of Bengal 40: 12–39.

[B2] Boulenger GA (1893) Concluding report on the reptiles and batrachians obtained in Burma by Signor L. Fea dealing with the collection made in Pegu and the Karin Hills in 1887–88. Annali del Museo Civico di Storia Naturale di Genova 13: 304–347.

[B3] Boulenger GA (1900) Descriptions of new batrachians and reptiles from the Larut Hills, Perak. Annals and Magazine of Natural History (Series 7) 6: 186–194. 10.1080/00222930008678356

[B4] Boulenger GA (1908) A revision of the Oriental pelobatid batrachians (genus *Megalophrys*). Proceedings of the Zoological Society of London 1908: 407–430. 10.1111/j.1096-3642.1908.tb01852.x

[B5] Bourret R (1937) Notes herpétologiques sur l’Indochine française. XIV. Les batraciens de la collection du Laboratoire des Sciences Naturelles de l’Université. Descriptions de quinze especes ou variétés nouvelles. Annexe au Bulletin Général de l'Instruction Publique, Hanoi 1937: 5–56.

[B6] Chen JM Poyarkov NJ Suwannapoom C Lathrop A Wu YH Zhou WW Yuan ZY Jin JQ Chen HM Liu HQ Nguyen TQ Nguyen SN Duong TV Eto K Nishikawa K Matsui M Orlov NL Stuart BL Brown RM Rowley J Murphy RW Wang YY Che J (2018) Large-scale phylogenetic analyses provide insights into unrecognized diversity and historical biogeography of Asian leaf-litter frogs, genus *Leptolalax*, (Anura: Megophryidae). Molecular Phylogenetics and Evolution 124: 162–171. 10.1016/j.ympev.2018.02.020 29530499

[B7] Chen W Liao XW Zhou SC Mo YM (2019) A new species of *Leptobrachella* (Anura: Megophryidae) from southern Guangxi, China. Zootaxa 4563(1): 67–82. 10.11646/zootaxa.4563.1.3 31716553

[B8] Dring J (1983) Frogs of the genus *Leptobrachella* (Pelobatidae). Amphibia–Reptilia 4(2): 89–102. 10.1163/156853883X00012

[B9] Dubois A (1983) Note préliminaire sur le genre *Leptolalax* Dubois, 1980 (Amphibiens, Anoures), avec diagnose d’une espèce novelle du Vietnam. Alytes 2: 147–153.

[B10] Dubois A (1987) Miscellanea taxinomica batrachologica (I). Alytes. Paris 5 [1986]: 7–95.

[B11] Delorme M Dubois A Grosjean S Ohler A (2006) Une nouvelle ergotaxinomie des Megophryidae (Amphibia, Anura). Alytes 24(1–4): 6–21.

[B12] Das I Tron RKL Rangad D Hooroo RN (2010) A new species of *Leptolalax* (Anura: Megophryidae) from the sacred groves of Mawphlang, Meghalaya, north-eastern India. Zootaxa 2339: 44–56. 10.11646/zootaxa.2339.1.2

[B13] Dehling JM (2012a) Eine neue Art der Gattung *Leptolalax* (Anura: Megophryidae) vom Gunung Benom, Westmalaysia/A new species of the genus *Leptolalax* (Anura: Megophryidae) from Gunung Benom, Peninsular Malaysia. Sauria 34: 9–21.

[B14] Dehling JM (2012b) Redescription of *Leptolalax gracilis* (Günther, 1872) from Borneo and taxonomic status of two populations of *Leptolalax* (Anura: Megophryidae) from Peninsular Malaysia. Zootaxa 3328: 20–34. 10.11646/zootaxa.3328.1.2

[B15] Dubois A Grosjean S Ohler A Adler K Zhao EM (2010) The nomenclatural status of some generic nomina of Megophryidae (Amphibia, Anura). Zootaxa 2493: 66–68. 10.11646/zootaxa.2493.1.6

[B16] Dehling JM Matsui M (2013) A new species of *Leptolalax* (Anura: Megophryidae) from Gunung Mulu National Park, Sarawak, East Malaysia (Borneo). Zootaxa 3670(1): 33–44. 26438919

[B17] Duong VT Do DT Ngo CD Nguyen TQ Poyarkov Jr (2018) A new species of the genus *Leptolalax* (Anura: Megophryidae) from southern Vietnam. Zoological Research 39(3): 181–196. 10.24272/j.issn.2095-8137.2018.009 PMC5968861 29643325

[B18] Edgar RC (2004) MUSCLE: multiple sequence alignment with high accuracy and high throughput. Nucleic Acids Research 32(5): 1792–1797. 10.1093/nar/gkh340 15034147 PMC390337

[B19] Eto K Matsui M Nishikawa K (2015) Description of a new species of the genus *Leptobrachella* (Amphibia, Anura, Megophryidae) from Borneo. Current Herpetology 34(2): 128–139. 10.5358/hsj.34.128

[B20] Eto K Matsui M Nishikawa K (2016) A new highland species of dwarf litter frog genus *Leptobrachella* (Amphibia: Anura: Megophryidae) from Sarawak. Raffles Bulletin of Zoology 64: 194–203.

[B21] Eto K Matsui M Hamidy A Munir M Iskandar D (2018) Two New Species of the Genus Leptobrachella (Amphibia: Anura: Megophryidae) from Kalimantan, Indonesia. Current Herpetology 37(2): 95–105. 10.5358/hsj.37.95

[B22] Fei L Ye CY (1990) Key to Chinese Amphibians. Publishing House for Scientific and Technological Literature, Chongqing, 364 pp. [in Chinese]

[B23] Fei L Ye CY (1992) The classification of Pelobatidae (*Leptolalax*) and a new species in China. Acta Zoologica Sinica 38(3): 245–253. [in Chinese]

[B24] Fei L Ye CY (2005) An Illustrated Key to Chinese Amphibians. Sichuan Publishing House of Science and Technology, Chongqing, 340 pp. [in Chinese]

[B25] Fouquet A Gilles A Vences M Marty C Blanc M Gemmell1 NJ (2007) Underestimation of species richness in Neotropical frogs revealed by mtDNA analyses. PLoS ONE 2(10): e1109. 10.1371/journal.pone.0001109 PMC2040503 17971872

[B26] Fei L Hu SQ Ye CY Huang YZ (2009) Fauna Sinica. Amphibia, Vol. 2, Anura. Science Press, Beijing, 957 pp. [in Chinese]

[B27] Fei L Ye CY Jiang JP (2012) Colored Atlas of Chinese Amphibians and yheir Distributions. Sichuan Science and Technology Press, Sichuan, 619 pp. [in Chinese]

[B28] Frost DR (2019) Amphibian Species of the World: an Online Reference. Version 6.0. Electronic Database. American Museum of Natural History, New York. http://research.amnh.org/herpetology/amphibia/index.html [Accessed on: 2019-8-13]

[B29] Günther A (1872) On the reptiles and amphibians of Borneo. Proceedings of the Zoological Society of London 1872: 586–600.

[B30] Günther A (1895) The reptiles and batrachians of the Natuna Islands. Novitates Zoologicae 2(4): 499–502.

[B31] Grismer LL Grismer JL Youmans TM (2004) A new species of *Leptolalax* (Anura: Megophryidae) from Pulau Tioman, West Malaysia. Asiatic Herpetological Research 10: 8–11.

[B32] Hu SQ Zhao EM Liu CC (1973) A survey of amphibians and reptiles in Kweichow province, including a herpetofaunal analysis. Acta Zoologica Sinica 19: 149–181.

[B33] Humtsoe LN Bordoloi S Ohler A Dubois A (2008) Rediscovery of a long known species, *Ixalus lateralis* Anderson, 1871. Zootaxa 1921: 24–34. 10.11646/zootaxa.1921.1.2

[B34] Hoang DT Chernomor O von Haeseler A Minh BQ Vinh L (2018) UFBoot2: improving the ultrafast bootstrap approximation. Molecular Biology and Evolution 35(2): 518–522. 10.1093/molbev/msx281 29077904 PMC5850222

[B35] Hou Y Zhang MF Hu F Li SY Shi SC Chen J Mo XY Wang B (2018) A new species of the genus *Leptolalax* (Anura, Megophryidae) from Hunan, China. Zootaxa 4444(3): 247–266. 10.11646/zootaxa.4444.3.2 30313922

[B36] Inger RF Stuebing RB (1992) A new species of frog of the genus *Leptobrachella* Smith (Anura: Pelobatidae), with a key to the species from Borneo. Raffles Bulletin of Zoology 39(1): 99–103.

[B37] Inger RF Stuebing RB Tan F (1995) New species and new records of anurans from Borneo. Raffles Bulletin of Zoology 43: 115–132.

[B38] Inger RF (1997) A new species of *Leptolalax* (Anura: Megophryidae) from Borneo. Asiatic Herpetological Research 7: 48–50. 10.5962/bhl.part.18855

[B39] Inger RF Orlov N Darevsky I (1999) Frogs of Vietnam: a report on new collections. Fieldiana, Zoology 92: 1–46. 10.5962/bhl.title.3478

[B40] Jiang K Yan F Suwannapoom C Chomdej S Che J (2013) A new species of the genus *Leptolalax* (Anura: Megophryidae) from northern Thailand. Asian Herpetological Research 4(2): 100–108. 10.3724/SP.J.1245.2013.00100

[B41] Kumar S Stecher G Tamura K (2016) MEGA7: Molecular Evolutionary Genetics Analysis Version 7.0 for Bigger Datasets. Molecular Biology and Evolution 33(7): 1870–1874. 10.1093/molbev/msw054 27004904 PMC8210823

[B42] Liu CC (1950) Amphibians of Western China. Fieldiana, America Zoology Memoires 2: 1–400. 10.5962/bhl.title.2977

[B43] Lathrop A Murphy RW Orlov N Ho CT (1998) Two new species of *Leptolalax* (Anura: Megophryidae) from northern Vietnam. Amphibia-Reptilia 19: 253–267. 10.1163/156853898X00160

[B44] Lanfear R Frandsen PB Wright AM Senfeld T Calcott B (2016) PartitionFinder 2: new methods for selecting partitioned models of evolution for molecular and morphological phylogenetic analyses. Molecular Biology and Evolution 34(3): 772–773. 10.1093/molbev/msw260 28013191

[B45] Malkmus R (1992) *Leptolalax pictus* sp. n. (Anura: Pelobatidae) vom Mount Kinabalu/Nord-Borneo. Sauria 14: 3–6.

[B46] Matsui M (1997) Call characteristics of Malaysian *Leptolalax* with a description of two new species (Anura: Pelobatidae) Copeia 1997: 158–165. 10.2307/1447851

[B47] Matsui M (2006) Three new species of *Leptolalax* from Thailand (Amphibia, Anura, Megophryidae). Zoological Science 23(9): 821–830. 10.2108/zsj.23.821 17043405

[B48] Matsui M Belabut DM Ahmad N Yong HS (2009) A new species of *Leptolalax* (Amphibia, Anura, Megophryidae) from peninsular Malaysia. Zoological Science 26(3): 243–247. 10.2108/zsj.26.243 19341347

[B49] Mathew R Sen N (2010) Description of a new species of *Leptobrachium* Tschudi, 1838 (Amphibia: Anura: Megophryidae) from Meghalaya, India. Records of the Zoological Survey of India 109: 91–108.

[B50] Matsui M Dehling J M (2012) Notes on an enigmatic Bornean megophryid, *Leptolalax dringi* Dubois, 1987 (Amphibia: Anura). Zootaxa 3317(1): 49–58. 10.11646/zootaxa.3317.1.4

[B51] Matsui M Zainudin R Nishikawa K (2014a) A new species of *Leptolalax* from Sarawak, western Borneo (Anura: Megophryidae). Zoological Science 31(11): 773–779. 10.2108/zs140137 25366161

[B52] Matsui M Nishikawa K Yambun P (2014b) A new *Leptolalax* from the mountains of Sabah, Borneo (Amphibia, Anura, Megophryidae). Zootaxa 3753(3): 440–452. 10.11646/zootaxa.3753.5.3 24869507

[B53] Mahony S Foley NM Bijumn SD Teeling EC (2017) Evolutionary history of the Asian horned frogs (Megophryinae): integrative approaches to timetree dating in the absence of a fossil record. Molecular Biology and Evolution 34(3): 744–771. 10.1093/molbev/msw267 28100792

[B54] Matsui M Eto K Nishikawa K Hamidy A Belabut D Ahmad N Panha S Khonsue W Grismer LL (2017) Mitochondrial phylogeny of *Leptolalax* from Malay Peninsula and *Leptobrachella* (Anura, Megophryidae). Current Herpetology 36(1): 11–21. 10.5358/hsj.36.11

[B55] Murphy RW Lathrop A Ho CT Orlov N (1998) Two new species of *Leptolalax* (Anura: Megophryidae) from northern Vietnam. Amphibia-Reptilia 19(3): 253–267. 10.1163/156853898X00160

[B56] Nguyen LT Schmidt HA Haeseler A von Minh BQ (2015) IQ-TREE: a fast and effective stochastic algorithm for estimating maximum-likelihood phylogenies. Molecular Biology and Evolution 32(1): 268–274. 10.1093/molbev/msu300 25371430 PMC4271533

[B57] Nguyen LT Poyarkov Jr NA Le DT Vo BD Phan HT Van DT Murphy WR Nguyen SN (2018) A new species of *Leptolalax* (Anura: Megophryidae) from Son Tra Peninsula, central Vietnam. Zootaxa 4388(1): 1–21. 10.11646/zootaxa.4388.1.1 29690461

[B58] Ohler A Marquis O Swan S Grosjean S (2000) Amphibian biodiversity of Hoang Lien Nature Reserve (Lao Cai Province, northern Vietnam) with description of two new species. Herpetozoa 13 (1/2): 71–87.

[B59] Ohler A Wollenberg KC Grosjean S Hendrix R Vences M Ziegler T Dubois A (2011) Sorting out *Lalos*: description of new species and additional taxonomic data on megophryid frogs from northern Indochina (genus *Leptolalax*, Megophryidae, Anura). Zootaxa 3147: 1–83. 10.11646/zootaxa.3147.1.1

[B60] Oberhummer E Barten C Schweizer IN Das I Haas AL Hertwig ST (2014) Description of the tadpoles of three rare species of megophryid frogs (Amphibia: Anura: Megophryidae) from Gunung Mulu, Sarawak, Malaysia. Zootaxa 3835: 59–79. 10.11646/zootaxa.3835.1.3 25081435

[B61] Poyarkov NA Rowley JJ Gogoleva SI Vassilieva AB Galoyan EA Orlov N L (2015) A new species of *Leptolalax* (Anura: Megophryidae) from the western Langbian Plateau, southern Vietnam. Zootaxa 3931: 221–252. 10.11646/zootaxa.3931.2.3 25781823

[B62] Rowley JJ Cao TT (2009) A new species of *Leptolalax* (Anura: Megophryidae) from central Vietnam. Zootaxa 2198(5): 51–60. 10.11646/zootaxa.2198.1.5 28610262

[B63] Rowley JJ Hoang DH Le TTD Dau QV Cao TT (2010a) A new species of *Leptolalax* (Anura: Megophryidae) from Vietnam and further information on *Leptolalax tuberosus*. Zootaxa 2660: 33–45.

[B64] Rowley JJ Stuart BL Neang T Emmett DA (2010b) A new species of *Leptolalax* (Anura: Megophryidae) from northeastern Cambodia. Zootaxa 2567: 57–68. 10.11646/zootaxa.2567.1.3 26624626

[B65] Rowley JJ Stuart BL Richards SJ Phimmachak S Sivongxay N (2010c) A new species of *Leptolalax* (Anura: Megophryidae) from Laos. Zootaxa 2681: 35–46. 10.11646/zootaxa.2681.1.3

[B66] Rowley JJ Le DTT Tran DTA Hoang DH (2011) A new species of *Leptobrachella* (Anura: Megophryidae) from southern Vietnam. Zootaxa 2796: 15–28. 10.11646/zootaxa.2796.1.2

[B67] Rowley JJ Hoang HD Dau VQ Le TTD Cao TT (2012) A new species of *Leptolalax* (Anura: Megophryidae) from central Vietnam. Zootaxa 3321: 56–68. 10.11646/zootaxa.3321.1.4

[B68] Ronquist F Teslenko M Van Der Mark P Ayres DL Darling A Höhna S Larget B Liu L Suchard MA Huelsenbeck JP (2012) MrBayes 3.2: efficient Bayesian phylogenetic inference and model choicem across a large model space. Systematic Biology 61: 539–542. 10.1093/sysbio/sys029 22357727 PMC3329765

[B69] Rowley JJ Dau VQ Nguyen TT (2013) A new species of *Leptolalax* (Anura: Megophryidae) from the highest mountain in Indochina. Zootaxa 3737(4): 415–428. 10.11646/zootaxa.3737.4.5 25112762

[B70] Rambaut A Suchard MA Xie D Drummond AJ (2014) Tracer v1. 6. http://beast.bio.ed.ac.uk/Tracer [Accessed on: 2019-3-7]

[B71] Rowley JJ Stuart BL Neang T Hoang HD Dau VQ Nguyen TT Emmett DA (2015a) A new species of *Leptolalax* (Anura: Megophryidae) from Vietnam and Cambodia. Zootaxa 4039: 401–417. 10.11646/zootaxa.4039.3.1 26624626

[B72] Rowley JJL Tran DTA Frankham GJ Dekker AH Le DTT Nguyen TQ Dau VQ Hoang HD (2015b) Undiagnosed Cryptic Diversity in Small, Microendemic Frogs (*Leptolalax*) from the Central Highlands of Vietnam. PLoS ONE 10 (5): e0128382. https://doi. org/10.1371/journal.pone.0128382 PMC4447284 26020250

[B73] Rowley JJ Tran DTA Le DTT Dau VQ Peloso PLV Nguyen TQ Hoang HD Nguyen TT Ziegler T (2016) Five new, microendemic Asian Leaf-litter Frogs (*Leptolalax*) from the southern Annamite mountains, Vietnam. Zootaxa 4085: 63–102. 10.11646/zootaxa.4085.1.3 27394289

[B74] Rowley JJ Dau VQ Hoang HD Le DTT Cutajar TP Nguyen TT (2017a) A new species of *Leptolalax* (Anura: Megophryidae) from northern Vietnam. Zootaxa 4243: 544–564. 10.11646/zootaxa.4243.3.7 28610143

[B75] Rowley J J Dau V Q Cao T T (2017b) A new species of *Leptolalax* (Anura: Megophryidae) from Vietnam. Zootaxa 4273(1): 61–79. 10.11646/zootaxa.4273.1.5 28610262

[B76] Smith MA (1925) Contributions to the herpetology of Borneo. Sarawak Museum Journal 3: 15–34.

[B77] Stejneger L (1926) Two new tailless amphibians from western China. Proceedings of the Biological Society of Washington 39: 53–54.

[B78] Smith MA (1931) The herpetology of Mt. Kinabalu, North Borneo, 13,455 ft. Bulletin of the Raffles Museum. Singapore 5: 3–32.

[B79] Simon C Frati F Beckenbach A Crespi B Liu H Flook P (1994) Evolution, weighting, and phylogenetic utility of mitochondrial gene sequences and a compilation of conserved polymerase chain reaction primers. Annals of the Entomological Society of America 87: 651–701. 10.1093/aesa/87.6.651

[B80] Sengupta S Sailo S Lalremsanga HT Das A Das I (2010) A new species of *Leptolalax* (Anura: Megophryidae) from Mizoram, north-eastern India. Zootaxa 2406: 56–68. 10.11646/zootaxa.2406.1.3

[B81] Sung YH Yang JH Wang YY (2014) A new species of *Leptolalax* (Anura: Megophryidae) from southern China. Asian Herpetological Research 5(2): 80–90. 10.3724/SP.J.1245.2014.00080

[B82] Taylor EH (1962) The amphibian fauna of Thailand. University of Kansas Science Bulletin 43: 265–599. 10.5962/bhl.part.13347

[B83] Watters JL Cummings ST Flanagan RL Siler CD (2016) Review of morphometric measurements used in anuran species descriptions and recommendations for a standardized approach. Zootaxa 4072(4): 477–495. 10.11646/zootaxa.4072.4.6 27395941

[B84] Wang J Yang JH Li Y Lyu ZT Zeng ZC Liu ZY Ye YH Wang YY (2018) Morphology and molecular genetics reveal two new *Leptobrachella* species in southern China (Anura, Megophryidae). ZooKeys 776: 105–106. 10.3897/zookeys.776.22925 PMC6072835 30100785

[B85] Wang J Li YL Li Y Chen HH Zeng YJ Shen JM Wang YY (2019) Morphology, molecular genetics, and acoustics reveal two new species of the genus *Leptobrachella* from northwestern Guizhou Province, China (Anura, Megophryidae). ZooKeys 848: 119–154. 10.3897/zookeys.848.29181 31160882 PMC6536485

[B86] Yang JH Wang YY Chen GL Rao DQ (2016) A new species of the genus *Leptolalax* (Anura: Megophryidae) from Mt. Gaoligongshan of western Yunnan Province, China. Zootaxa 4088: 379–394. 10.11646/zootaxa.4088.3.4 27394346

[B87] Yuan ZY Sun RD Chen J Rowley JJ Wu ZJ Hou SB Wang SN Che J (2017) A new species of the genus *Leptolalax* (Anura: Megophryidae) from Guangxi, China. Zootaxa 4300(4): 551–570. 10.11646/zootaxa.4300.4.5

[B88] Yang JH Zeng ZC Wang YY (2018) Description of two new sympatric species of the genus *Leptolalax* (Anura: Megophryidae) from western Yunnan of China. PeerJ 6: e4586. 10.7717/PeerJ.458 PMC5898428 29666755

